# The stromal morphological changes for differential diagnosis of uninodular high-grade dysplastic nodule and well-differentiated small hepatocellular carcinoma

**DOI:** 10.18632/oncotarget.20607

**Published:** 2017-09-01

**Authors:** Long-Hai Feng, Han Wang, Hui Dong, Yu-Yao Zhu, Wen-Ming Cong

**Affiliations:** ^1^ Department of Pathology, Eastern Hepatobiliary Surgery Hospital, The Second Military Medical University, Yangpu, Shanghai, 200438, China; ^2^ Key Laboratory of Signaling Regulation and Targeting Therapy of Liver Cancer (Second Military Medical University) Ministry of Education, Yangpu, Shanghai, 200438, China; ^3^ Shanghai Key Laboratory of Hepatobiliary Tumor Biology (Eastern Hepatobiliary Surgery Hospital), Yangpu, Shanghai, 200438, China

**Keywords:** HGDNs, WD-SHCCs, stromal changes, diagnosis, differential diagnosis

## Abstract

**Aim:**

The stromal invasion has been regarded as the most valuable clue to distinguish high-grade dysplastic nodules (HGDNs) and well-differentiated small hepatocellular carcinomas (WD-SHCCs). The purposes of this study are to explore the stromal morphological changes for the differential diagnosis of these two equivocal lesions.

**Results:**

Based on the systemic studies of histological characteristics of HGDNs and WD-SHCCs, the stromal morphological changes, including sinusoid capillarization, ductular reaction and solitary artery, were performed to make a differential diagnosis between them. Separately, the solitary artery had the best sensitivity (93.75%) and accuracy (88.89%), and the sinusoid capillarization had the best specificity of 90.32%. On the whole, when at least 2 stromal morphological changes were abnormal, no matter what combination, the diagnostic performance was favorable and optimal with the highest accuracy of 92.06%, balancing the sensitivity (93.75%) and specificity (90.32%). The diagnostic performances were prior to the classical immunohistochemical panel comprising heat shock protein 70, glypican 3 and glutamine synthetase with the best sensitivity, specificity and accuracy of 62.50%, 80.65% and 71.43%, respectively.

**Materials and Methods:**

A retrospective case-control study was conducted on 63 patients who underwent partial hepatectomy for uninodular HGDNs or WD-SHCCs at the Eastern Hepatobiliary Surgery Hospital from 2005 to 2015.

**Conclusions:**

The stromal morphological changes, containing sinusoid capillarization, ductular reaction and solitary artery could provide a more considerable diagnostic and differential diagnostic performance between HGDNs and WD-SHCCs. And they should be the key points during the histopathological diagnosis.

## INTRODUCTION

High-grade dysplastic nodules (HGDNs) and well-differentiated small hepatocellular carcinomas (WD-SHCCs) are the most significant stages in the multistep pathogenesis ranging from low-grade dysplastic nodules (LGDNs), HGDNs and WD-SHCCs to advanced HCC [1]. The differential diagnosis of HGDNs and WD-SHCCs continues to be difficult even for experienced pathologists.

Over the years, outstanding progress has been achieved in understanding these two ambiguous nodules [2–5]. First, the characteristics of cell and stromal morphological changes (SMCs) have achieved a consensus. Second, stromal invasion is now regarded as the most valuable clue to distinguish WD-SHCCs from HGDNs. Third, nodule in nodule (NIN) is an important mechanism of cancerization in HGDNs. Fourth, the classical immunohistochemical markers, including heat shock protein 70 (HSP70), glypican 3 (GPC3) and glutamine synthetase (GS), have been verified as a rather effective panel for WD-SHCCs by multiple clinical trials in both surgically resected samples and liver biopsies [6–10]. Finally, some other novel panels with much better diagnostic performance have been proposed, but they have not been verified by multi-center research studies [11–13].

In this study, we will mainly focus on the SMCs of uninodular HGDNs and WD-SHCCs, especially sinusoid capillarization (SC), ductular reaction (DR) in marginal area of tumor and solitary artery (SA). Traditionally, SC was considered to reflect the dedifferentiation of the liver tissue during the course of cirrhosis [14]. It can be assessed by cluster of differentiation 34 (CD34), a classical vascular endothelial cell marker, directly. DR in marginal areas or within the nodule is an effective indicator to estimate whether stromal invasion exists [15]. And stromal invasion, in which tumor cells invade the fibrous tissue of portal tracts within or outside the nodule, has been regarded as the most valuable clue for diagnosis of WD-SHCCs. DR could be immunostained with cytokeratin 19 or cytokeratin 7 (CK19/CK7), a favorable marker for small bile ducts [16]. SA, also known as unpaired arteries, is defined as an isolated artery without a corresponding concomitant bile duct. It usually has a regular, stretchy lumen and thick vascular wall with serrated arranged endothelial nuclei. SA is a reflection of neovascularization, and its density increases gradually from LGDNs to HGDNs to WD-SHCCs [3]. However, for the differential diagnosis of HGDNs and WD-SHCCs, its diagnostic value has not been estimated. SA can be detected on the Hematoxylin and Eeosin (HE) staining directly.

Consequently, the purposes of this study are to characterize the clinicopathological features of uninodular HGDNs and WD-SHCCs, to explore the stromal changes, SC, DR and SA, for the differential diagnosis of these two equivocal lesions.

## RESULTS

### Clinicopathological features

According to the inclusion and exclusion criteria, of 66 patients with HGDNs who received partial hepatectomy during the study period, only 31 were brought into the study. Of the 3000 patients diagnosed with SHCCs, only 32 meet the inclusion criteria. The detailed clinicopathological features of these uninodular HGDNs and WD-SHCCs are listed in Table 1, and the primary clinical information of each patient is shown in Supplementary Tables 1 and 2. Notably, NIN was identified in 15 of 31 HGDNs (48.39%), and 14 nodules (43.75%) did not have a complete IPA among these 32 WD-SHCCs.

**Table 1:** Clinicopathological features of uninodular HGDNs and WD-SHCCs

Clinicopathological parameters	HGDNs (*n* = 31)	WD-SHCCs (*n* = 32)	*P* Value
Sex			
Female	9 (29.03%)	7 (21.88%)	0.514
Male	22 (70.97%)	25 (78.12%)	
Age	56.61 ± 7.45	57.66 ± 9.23	0.624
Tumor Size (cm)	2.63 ± 1.00	2.14 ± 0.61	0.021
Hepatitis			
HBV or HCV	31 (100%)	28 (87.50%)	0.113
None	0 (0.00%)	4 (12.50%)	
Platelet (×10^9^/L)			
< 125	27 (87.10%)	13 (63.16%)	< 0.001
125–350^*^	4 (12.90%)	19 (36.84%)	
Prothrombin time (sec.)	13.26 ± 1.42	12.27 ± 1.15	0.008
Total bilirubin (μmol/L)			
5.1–18.8^*^	20 (64.52%)	25 (78.13%)	0.232
> 18.8	11 (35.48%)	7 (21.87%)	
Albumin (g/L)	38.60 ± 3.91	40.53 ± 4.82	0.087
Globulin (g/L)	31.31 ± 4.83	29.02 ± 5.13	0.073
Albumin / Globulin	1.27 ± 0.25	1.44 ± 0.29	0.012
Alanine aminotransferase (U/L)			
0–41^*^	16 (51.61%)	25 (78.13%)	0.027
> 41	15 (48.39%)	7 (21.87%)	
Aspartate aminotransferase (U/L)			
0–37^*^	17 (54.84%)	23 (71.18%)	0.160
> 37	14 (45.16%)	9 (28.12%)	
Alkaline phosphatase (U/L)			
40–129^*^	26 (83.87%)	25 (78.13%)	0.561
> 129	5 (16.13%)	7 (21.87%)	
Glutamyltransferase (U/L)			
0–41 (Female)^*^ or 0–61 (Male)^*^	20 (64.52%)	13 (40.63%)	0.058
> 41 (Female) or > 61 (Male)	11 (35.48%)	19 (59.37%)	
Alpha fetal protein (μg/l)			
0–20^*^	20 (64.52%)	30 (93.75%)	0.004
>20	11 (35.48%)	2 (6.25%)	
Carbohydrate antigen 19-9 (U/ml)			
0–39^*^	11 (36.67%)	26 (81.25%)	< 0.001
> 39	20 (63.33%)	6 (18.75%)	
Carcinoembryonic antigen (U/ml)			
0–10^*^	30 (96.77%)	28 (87.50%)	0.355
> 10	1 (3.23%)	4 (12.50%)	
Cirrhosis			
Yes	26 (83.87%)	16 (50%)	0.004
No	5 (16.13%)	16 (50%)	
Pseudoglands structures			
Yes	16 (51.61%)	13 (40.63%)	0.382
No	15 (48.39%)	19 (59.37%)	
Steatosis			
Yes	26 (83.87%)	21 (65.63%)	0.096
No	5 (16.13%)	11 (34.37%)	
Sinusoid capillarization			
Low	28 (90.32%)	7 (21.88%)	< 0.001
Mild or serious	3 (10.71%)	25 (78.12%)	
Ductular reaction			
Consecutiveness	25 (80.65%)	4 (12.50%)	0.0001
Interruption or absence	6 (19.35%)	28 (87.50%)	
Solitary arteries			
0–2/10 MPFs	26 (83.87%)	2 (6.25%)	< 0.001
≥ 2/10 MPFs	5 (16.13%)	30 (93.75%)	
Solitary arteries			
0–1/ 1 MPF	26 (83.87%)	8 (25%)	< 0.001
≥ 2/ 1 MPF	5 (16.13%)	24 (75%)	
Intranodule portal area†			
0–1/10 MPFs	3 (9.68%)	29 (90.63%)	< 0.001
≥ 1/10 MPFs	28 (90.32%)	3 (9.37%)	

HGDNs, high-grade dysplastic nodules; WD-SHCCs, well-differentiated small hepatocellular carcinomas; \, none; ^*^, normal reference value; †14 WD-SHCCs had no intranodule portal area.

**Table 2:** Diagnostic evaluation of SMCs for WD-SHCCs detection

Subgroups	WD-SHCCs (*n* = 32)	HGDNs (*n* = 31)	Sensitivity (%)	Specificity (%)	PPV (%)	NPV (%)	Accuracy (%)
3 Changes							
All 3 positive	21	0	65.63	100.00	100.00	73.81	82.54
At least 2 positive	30	3	93.75	90.32	90.91	93.33	92.06
At least 1 positive	32	11	100.00%	64.52	74.42	100.00	82.54
2 Changes							
SC+ and DR+	22	0	68.75	100.00	100.00	75.61	84.13
SC+ and SV+	24	1	75.00	96.77	96.00	78.95	85.71
DR+ and SV+	26	2	81.25	93.55	92.86	82.86	87.30
1 Change							
SC+	25	3	78.13	90.32	89.29	80.00	84.13
DR+	28	6	87.50	80.65	82.35	86.21	84.13
SA+	30	5	93.75	83.87	85.71	92.86	88.89

Abbreviations: WD-SHCCs, well-differentiated small hepatocellular carcinomas; HGDNs, high-grade dysplastic nodules; PPV: positive predictive value; NPV: negative predictive value; SC, sinusoid capillarization; DR, ductular reaction; SV, solitary vessel.

As shown in Table 1, there were significant differences between HGDNs and WD-SHCCs in multiple preoperative clinicopathological parameters, including tumor sizes, platelets (PLT), prothrombin time (PT), albumin/globulin (A/G), alanine aminotransferase (ALT), α-fetal protein (AFP), carbohydrate antigen19-9 (CA19-9), cirrhosis, SC, DR, SA and IPA. A multivariate analysis by Logistic Regression had been made to exclude the interaction of SC, DR and SA. It is indicated that these three factors had significant difference between HGDNs and WD-SHCCs with similar odd ratios (Supplementary Table 5).

### Stromal morphological changes

#### Sinusoid capillarization

**Figure 1:**
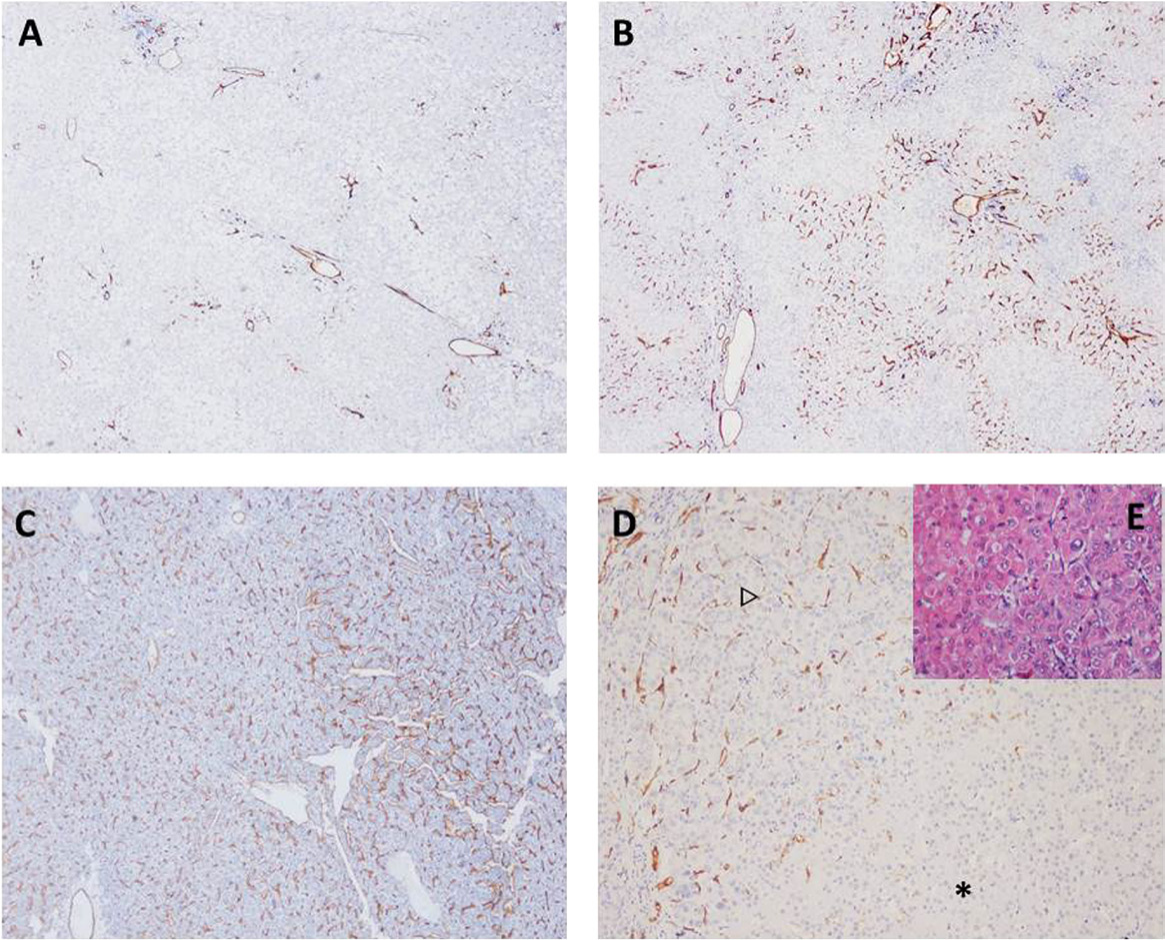
The classifications of sinusoid capillarization (SC) and immunohistochemical staining of CD34 (**A**) CD34, low expression, multifocal positive without bridging or fusion (×40, HGDNS); (**B**) CD34, mild expression, positive with bridging or fusion (×40, HGDNs); (**C**) CD34, severe expression, diffuse positive (×40, WD-SHCCs); (**D**) CD34, severe expression in nodule in nodule (×100, ^*^, HGDNs; △, nodule in nodule); (**E**) histological changes in the junction area of HGDNs and NIN (hematoxylin-eosin staining, ×200).

Based on the immunostaining of CD34, capillarized sinusoid was observed in all 63 cases. In the HGDNs cohort, 28 (90.32%) nodules presented low SC, 2 (6.45%) mild SC and 1 (3.13%) severe SC. These percentages in the WD-SHCCs group were 21.88% (7/32), 12.50% (4/32) and 65.62% (21/32), respectively. Moreover, severe SC could appear in the NIN of HGDNs (Figure 1). Overall, the sensitivity, specificity, positive predictive value (PPV), negative predictive value (NPV) and accuracy for WD-SHCCs diagnosis were 78.13%, 90.32%, 89.29%, 80.00% and 84.13%, respectively.

### Ductular reaction

**Figure 2:**
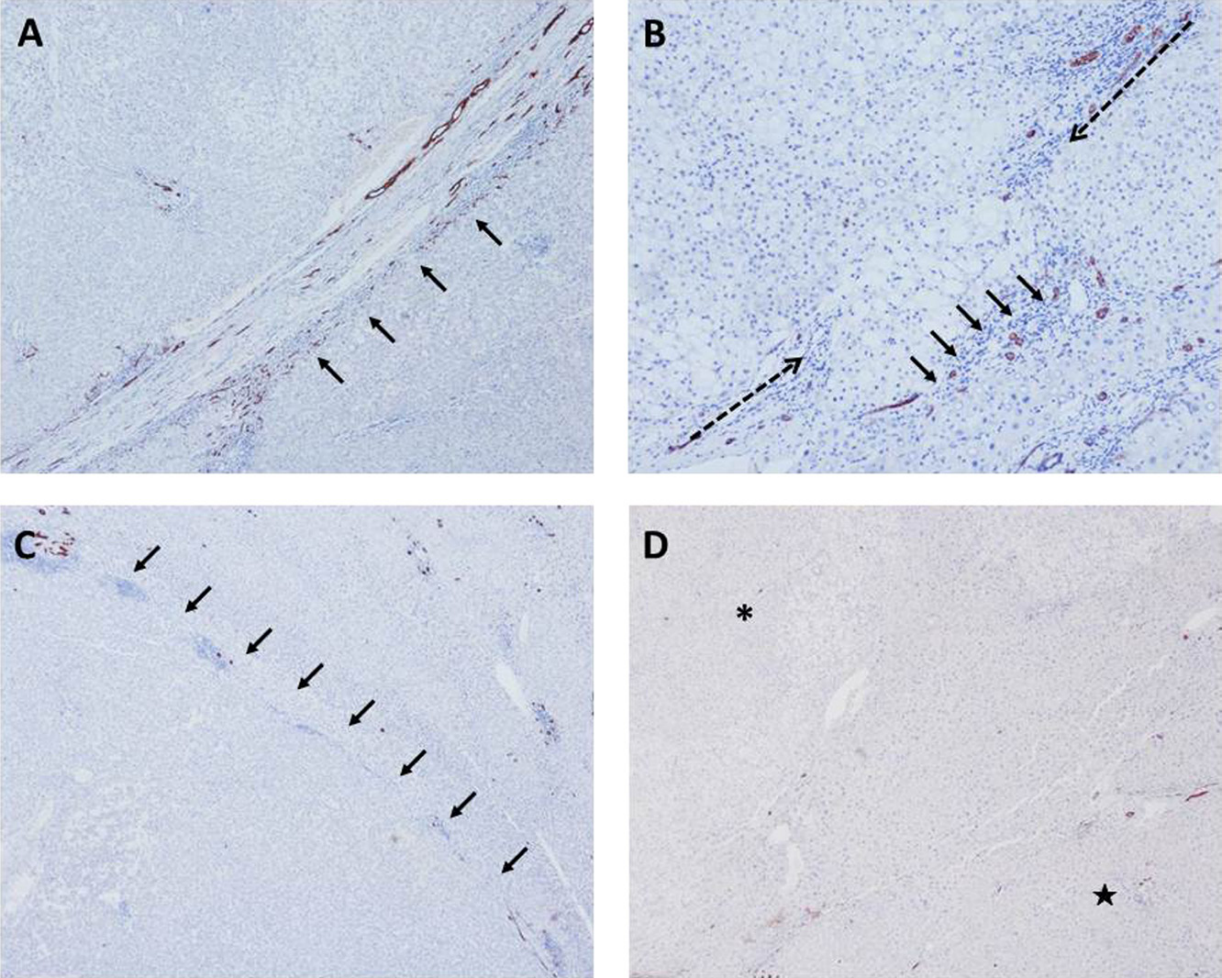
The classifications of ductular reaction (DR) and immunohistochemical staining of CK19 in the margin of nodule (arrows) (**A**) CK19, consecutive DR (× 100, HGDNs); (**B**) discontinuous DR, (×100, WD-SHCCs); (**C**) absent DR in the margin of the nodule, CK19 negative (× 40, WD-SHCCs). (**D**) absent DR in the transition region of nodule without intact fibrous capsule (× 40, ^*^, HGDNs; ★, normal liver tissue).

According to the immunoreactivity of CK19, consecutive DR in the marginal area of nodule was observed in 25 of 31 (80.65%) HGDNs, the others (19.35%) were all inconsecutive (Figure 2). In WD-SHCCs cohort, consecutive DR emerged in 4 nodules (12.50%), the inconsecutive presented in 17 nodules (53.13%) and the other 11 nodules (34.37%) were absent. Overall, the values of sensitivity, specificity, PPV, NPV and accuracy for WD-SHCCs diagnosis were 87.50%, 80.65%, 82.35%, 86.21% and 84.13%, respectively.

### Solitary artery

**Figure 3:**
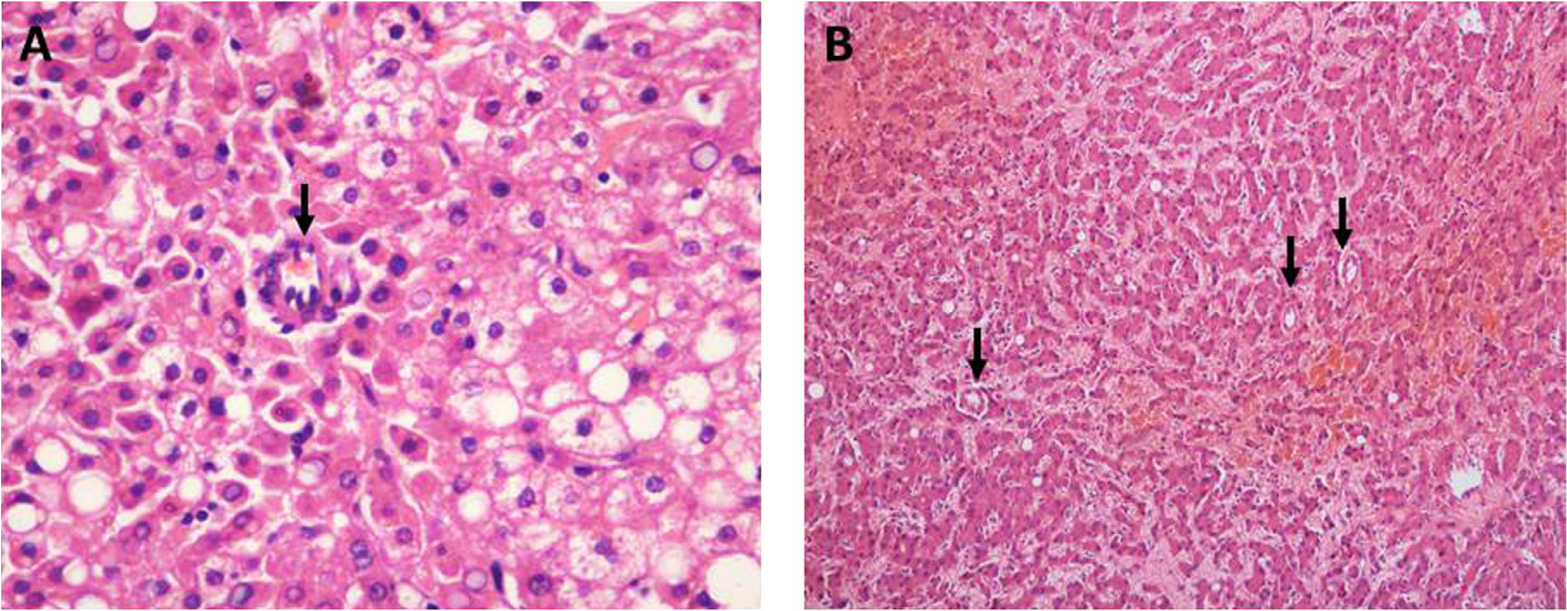
The solitary artery (SA, the arrowhead points) in routine hematoxylin-eosin (HE) staining (**A**) in the area of steatosis, SA was usually rare with a narrow lumen (×400, HGDNs); (**B**) multi-arteries were observed in only one view (×100, WD-SHCCs).

SA was observed in all nodules (Figure 3). The average density was 1.26/10 MPFs in HGDNs, with a range of 0.33 to 4.17, as opposed to 9.19/10 MPFs in WD-SHCCs, with a range of 0.95 to 21.63. The average densities of 26 HGDNs (83.87%) were no more than 2/10 MPFs. In contrast, 93.75% of WD-SHCCs were greater than 2/10 MPFs. In one view of MPF, 75% of WD-SHCCs had at least 2 SAs, and it was only observed in 16.13% of HGDNs.

SAs were scarce in the steatosis area, and compared to the HGDNs, the density did not change notably in NIN of HGDNs. Overall, the sensitivity, specificity, PPV, NPV and accuracy of the average density of SA for detection of WD-SHCCs were 93.75%, 83.87%, 85.71%, 92.86% and 88.89%, respectively.

### Combination of SC (marked by CD34), DR (marked by CK19) and SA in the differential diagnosis between HGDNs and WD-SHCCs

The 8 potential combinations of SMCs in HGDNs and WD-SHCCs are summarized in Supplementary Table 3. The panels SC+/DR+/SA+ (all 3 positive) and SC+/DR+/SA– were only observed in WD-SHCCs, with a proportion of 65.62% and 3.13%, respectively. Conversely, the combinations SC–/DR–/SA– (all 3 negative) and SC+/DR–/SA– were only present in 64.52% and 6.45% of HGDNs, respectively, but not in WD-SHCCs. The other combinations emerged in HGDNs and WD-SHCCs with a limited proportion.

Based on the potential combinations, the differential diagnosis of SMCs between HGDNs and WD-SHCCs was classified and performed independently as follows: (1) all 3 SMCs positive; (2) at least 2 SMCs positive (4 potential combinations in total); (3) at least 1 SMC positive (7 potential combinations in total). The diagnostic performances of SMCs for WD-SHCCs are enumerated in Table 2. The best sensitivity (100%) was acquired when at least 1 SMC was positive, but with an insufficient specificity (64.52%). Similarly, with the specificity of 100.00% (all 3 positive), the sensitivity dropped to 65.63%. However, these two criteria had the same accuracy of 82.54%. On the whole, when at least 2 SMCs were positive, no matter what combination, the diagnostic performance was favorable and optimal with the highest accuracy of 92.06%, balancing the sensitivity and specificity. In addition, only 2 or 1 SMC for differential diagnosis was assessed simultaneously (Table 2).

### Classical parenchymal panel in the differential diagnosis between HGDNs and WD-SHCCs

The immunohistochemical staining for HSP70, GPC3 and GS had been reported partially before, and the results of diagnostic efficacy were summarized in Table 3 [17]. Overall, when at least 2 of them were positive, the differential diagnosis effect for WD-SHCCs was slightly better than the others.

**Table 3:** Diagnostic evaluation of GPC3, HSP70 and GS for WD-SHCCs detection

Subgroups	WD-SHCCs (*n* = 32)	HGDNs (*n* = 31)	Sensitivity (%)	Specificity (%)	PPV (%)	NPV (%)	Accuracy (%)
3 Markers							
All 3 positive	5	0	15.65	100.00	100.00	53.44	57.14
At least 2 positive	20	6	62.50	80.65	76.92	67.57	71.43
At least 1 positive	30	17	93.75	45.16	63.83	87.50	69.84
2 Markers							
HSP70+ and GS+	17	4	53.13	87.10	80.95	64.29	69.84
HSP70+ and GPC3+	8	1	25.00	96.77	88.89	55.56	60.32
GPC3+ and GS+	5	1	15.63	96.77	83.33	52.63	55.56
1 Marker							
HSP70+	26	10	81.25	67.74	72.22	77.78	74.60
GS+	21	9	65.63	70.97	70.00	66.67	68.25
GPC3+	8	4	25.00	87.10	66.67	49.09	55.56

Abbreviations: WD-SHCCs, well-differentiated small hepatocellular carcinomas; HGDNs, high-grade dysplastic nodules; PPV: positive predictive value; NPV: negative predictive value; HSP70, heat shock protein 70; GPC3, glypican 3; GS, glutamine synthetase; +, positive; -, negative.

## DISCUSSION

The equivocal nodules, HGDNs and WD-SHCCs, have common pathologic cytological changes and architectural disturbances including small cell change (SCC), cytoplasmic basophilia, nuclear abnormalities and irregularities, cell crowding, steatosis, thickening hepatocyte plate, pseudoglands, solitary arteries, capillarized sinusoids, reticulin framework and stromal invasion. These analogical and complex changes make it difficult to distinguish one from the other. Novel molecular markers containing sulfite oxidase, aldo-ketoreductase family 1 member B10 and leukemia inhibitory factor receptor have achieved significant progress [10, 15]. In this study, CD34 and CK19 were selected as the markers for SC and DR, which demonstrate the degree of differentiation and stromal invasion. SA, presenting the neovascularization, was observed and assessed quantificationally to distinguish HGDNs and WD-SHCCs from each other.

Based on strict inclusion and exclusion criteria, 31 uninodular HGDNs and 32 WD-SHCCs without any other concomitant malignant lesions were screen out. Then the clinicopathological data were analyzed systematically and synthetically. In these two groups of patients, we also found some unexpected preoperative clinicopathologic manifestations. First, all of the patients had a Child-Pugh classification of A or B, but they were significantly different in the levels of PLT, PT, A/G and ALT, which seemed more unfavorable in the HGDNs patients than the WD-SHCCs. Second, the serological tumor markers AFP and CA19-9 were also significantly different between them. Likewise, most HGDNs patients had a higher level of AFP or CA19-9 than WD-SHCCs. These results suggest that negative serum AFP or CA19-9 is a distinctive feature of WD-SHCCs rather than HGDNs. Third, among the HGDNs cohort, the levels of AFP and CA19-9 did not correlate with the NIN (*P*_AFP_ = 1.00, *P*_CA19-9_= 1.00). Fourth, a diagnostic evaluation of hematic PLT, AFP and CA19-9 for HGDNS detection was also operated to access if they could make a differential diagnosis between them (Supplementary Table 4). Individually, PLT had the highest sensitivity, 83.87%, and AFP had the highest specificity, 93.75%. When at least 2 of these parameters were abnormal, the diagnosis effect for HGDNs is slightly better than the others and is similar to the classical parenchymal panel for WD-SHCCs, even slightly better. This might have accessory diagnostic value for preoperative diagnosis or liver puncture diagnosis due to the equivocal nodules. These features have never been reported before. The pathological mechanism of them still need further study.

CD34, a marker related to dedifferentiation of tumor cell, has a favorable effect in differential diagnosis between HGDNs and WD-SHCCs [10, 18]. Most HGDNs expressed CD34 focally, but not diffusely, in contrast to WD-SHCCs. The mild SC did not have a large proportion in HGDNs or WD-SHCCs. And in the NIN of HGDNs, CD34 was usually expressed diffusely (Figure 1). These data demonstrate the differences of cellular differentiation degrees between HGDNs and WD-SHCCs. Importantly, 21.88% (7/32) of WD-SHCCs nodules presented low SC.

DR, marked by CK19, has been regarded as an effective marker for stromal invasion. It implies a reaction of the ductular phenotype that may arise from proliferation of pre-existing cholangiocytes, progenitor cells or biliary metaplasia of hepatocytes [19]. It is often absent in minimally invasive and overtly invasive HCC, which makes it a helpful method to confirm whether stromal invasion exists. Because the marginal area of HCC is regarded as a representative region of tumor heterogeneity, a region with a high concentration of highly aggressive tumor cells, and a high-risk region related to recurrence or metastasis, the DR in the marginal area of nodule instead of IPA was chosen as a main evaluation indicator of stromal invasion [20–21]. Consecutive DR was observed in most HGDNs (80.65%) and a minority of WD-SHCCs (12.50%). Small bile ducts immunostained by CK19 were arranged in a line without interruption or absence in these nodules. In most WD-SHCCs, the interruption or absence of DR was more frequent, indicating the presence of stromal invasion. In the discontinuous interval of small bile ducts, CK19 was negative or positive sporadically, and the small bile ducts were arranged outwardly. However, in the nodules with a transition region from small cell changes to normal hepatic cells, CK19 was often negative. And these nodules often had no or incomplete fibrous capsules (Figure 2).

SA (also named as unpaired artery) is a new supplying artery without corresponding concomitant bile duct. Combined with the portal area within the nodule, it constitutes a double blood supply in the nodule, which has an important effect on the development and progression of a tumor. Traditionally, the number and density of SAs increase gradually from LGDNs to HGDNs and WD-SHCCs. Indeed, we found it had a significant difference between HGDNs and WD-SHCCs, whether analyzing the average density (per 10 MPFs) or one MPF. Multi-solitary arteries were frequently located in only one MPF in WD-SHCCs, but it was extremely rare in HGDNs (Figure 3). SA was also scarce in the steatotic region. Maybe the fatty degeneration occurred because of the lack of SA, which induced ischemia and anoxia.

In the NIN of HGDNs, SA was still infrequent, but SC increased significantly, demonstrating that capillarized sinusoid probably occurred earlier than neovascularization during the carcinogenic process from HGDNs to WD-SHCCs. On imaging, HGDNs usually appears hypervascular or isovascular, but hypovascularity can also emerge in WD-SHCCs [3]. This might be determined by the differences of SA or blood supply between them.

The diagnostic value for WD-SHCCs of the stromal changes had never been evaluated synthetically before. Individually, CD34 had the best specificity of 90.32%, and SA had the top sensitivity of 78.13%. Among the pairwise combinations, CK19 and SA had the highest positive rate (81.25% in WD-SHCCs and 6.45% in HGDNs). Overall, when at least 2 of them were positive, the diagnostic effect was the best, with sensitivity, specificity, PPV, NPV and accuracy of 93.75%, 90.32%, 90.91%, 93.33% and 92.06%, respectively. All 3 positive was only observed in WD-SHCCs.

The classical immunohistochemical panel of HSP70, GPC3 and GS was also performed and evaluated (Table 3). Individually, HSP70 had the highest positive rate (81.25%) in WD-SHCCs, followed by GS (65.63%) and GPC3 (25.00%). The expression of HSP70 and GS was significantly different between WD-SHCCs and HGDNs (P_HSP70_ < 0.001, P_GS_ < 0.004), but not GPC3 (P_GPC3_ < 0.222). That means GPC3 does not have sufficient diagnostic efficiency to distinguish WD-SHCCs from HGDNs. This might be because GPC3 immunoreactivity was affected by the tumor differentiation grade. Synthetically, when at least 2 markers were positive, the diagnostic performance for WD-SHCCs was slightly better than the others. In previous reports, the sensitivity of this panel for WD-SHCCs varied from 33.3% to 71.9%, but with an identical specificity of 100.00% [6–10]. In our cases, the specificity decreased to 80.65%, which was mainly caused by the low expression rates of HSP70 and GS in HGDNs. Anyway, we believe that the differential diagnostic efficiency for HGDNs and WD-SHCCs could be improved if it is combined with SMCs and immunohistochemical examinations.

Remarkable advances, especially on contrast-enhanced multiphasic computed tomography (CT) or magnetic resonance imaging (MRI), have been made recently in the imaging diagnosis for different types of liver lesions [22]. We also extract the imaging data of our patients. There were 25 of 31 HGDNs and 29 of 32 WD-SHCCs patients who had contrast-enhanced CT or MRI. According to the guidelines of the European Association for the Study of the Liver (EASL) and the American Association for the Study of Liver Disease (AASLD), the diagnostic performance of imaging for WD-SHCCs detection has been made (Supplementary Table 6) [23–24]. The sensitivity, specificity, PPV, NPV and accuracy of imaging for WD-SHCCs were 59.01%, 70.00%, 89.66%, 28.00% and 61.11%, respectively. Overall, the performance of hematic PLT, AFP and CA19-9 might be prior the imaging on the differential diagnosis between HGDNs and WD-SHCCs.

There are several limitations to this study. First, the number of cases is small due to its scarcity, even though it is the largest resected HGDNs study so far reported in a single center. Second, the present study is a retrospective clinicpathological cohort study. So far, only one prospective study on HGDNs has been reported [25], in which, 19 HGDNs were confirmed by liver biopsies and were followed-up by ultrasound and serum alfa-fetoprotein determination for assessing the incidence of HCC development [25]. Further we will perform a prospective controlled study to validate the value of SMCs in differential diagnosis of HGDNs and WD-SHCCs.

In conclusion, based on the systemic studies of histological characteristics of HGDNs and WD-SHCCs, the SC, DR and SA, reflecting the dedifferentiation degree, stromal invasion, and neovascularization of a nodule lesion, had a favorable diagnostic value for WD-SHCCs. The diagnostic effects were superior to the classical immunohistochemical panel of HSP70, GPC3 and GS. These changes should attract more attentions during the histopathological examinations. The serological changes of PLT, AFP and CA19-9 might have accessory diagnostic value for preoperative diagnosis or liver puncture diagnosis due to the equivocal nodules.

## MATERIALS AND METHODS

### Clinical material

A retrospective case-control study was conducted on two cohorts of patients who underwent curative resection at the Eastern Hepatobiliary Surgery Hospital (EHBH, Shanghai, China) and were ultimately diagnosed with HGDNs or WD-SHCCs (including small hepatocellular carcinoma grade 1 (SHCC-G1), based on the Edmondson and Steiner criteria) between July 2005 and March 2015.

Inclusion criteria were as follows: (1) single nodule; (2) the liver nodule was first detected; (3) the pathological diagnosis of HGDNs or WD-SHCCs was first diagnosed definitely; (4) operation was the first treatment; (5) diameter of WD-SHCCs ≤ 3 cm. Exclusion criteria included the following: (1) multiple nodules; (2) recurrent HGDNs or WD-SHCCs; (3) previous medical history of hepatic or other malignant tumor resection; (4) suffering from hepatic schistosomiasis.

**Figure 4:**
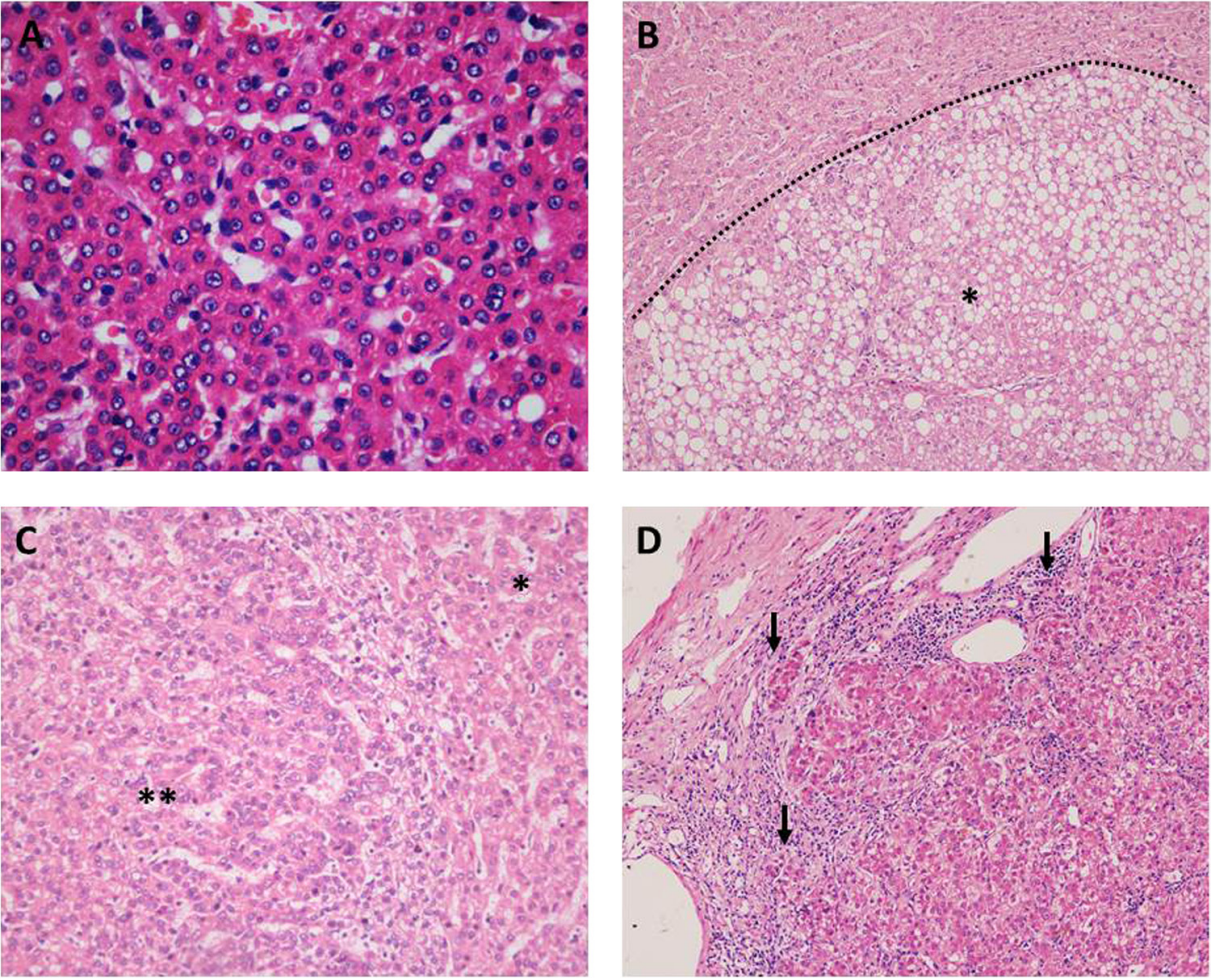
The histomorphological changes of HGDNs and WD-SHCCs (hematoxylin-eosin staining) (**A**) small cell change (×400); (**B**) small cell change, steatosis and bulging clonal growth (dotted line, ×100, ^*^, HGDNs); (**C**) nodule in nodule (^**^) of HGDNs (^*^), more cytological atypia and architectural disturbances (×200); (**D**) stromal invasion in WD-SHCCS (arrows, ×100).

The pathological diagnoses were operated by the committee which was consisted of multiple experienced pathologists and based on the updated criteria of the WHO classification of the digestive system and the latest international consensus of nodular lesions in cirrhotic liver [26–27] (Figure 4). The preoperative clinicopathological data, including initial liver disease, clinical manifestation, blood routine examination, coagulation and liver function, the levels of tumor markers, imaging performance, and cellular and stromal morphological changes, were extracted from each archive.

### Specimen preparation and immunostaining

The surgical specimens were sampled with surrounding liver tissues integrally and dehydrated using the Leica ASP300S Fully Enclosed Tissue Processor (Leica, Darmstadt, Germany) for 12 hours. The specimens were subsequently embedded in paraffin, and 4-μm-thick sections were cut and stained with HE for routine light microscopy examination. Then, a typical section was selected to perform immunostaining with an automated immunostainer (Leica BOND-MAX™, Darmstadt, Germany). The antibodies applied in our study were CD34 (dilution, 1:300), CK19 (dilution, 1:400), GPC3 (dilution, 1:300), HSP70 (dilution, 1:200), and GS (1:500). The HSP70 and GS antibodies were purchased from Maixin Biotech (Fuzhou, China), the others from Changdao (Shanghai, China). Based on our own experience, CK19 was used to distinguish DR in this study because of its higher sensitivity than other biliary markers.

### Evaluation

All cases were observed and reviewed by two experienced pathologists. If there was any disagreement, it would be settled by the committee of pathologists. The degree of SC was assessed based on the immunohistochemical characteristics of CD34 and was further subclassified as below: low = focal or multifocal positive without bridging or fusion (positive area <10%); mild = multifocal positive with bridging or fusion positive (positive area = 10%–50%); severe = diffuse positive (positive area > 50%, Figure 1). The higher its classification was, and the less differentiated it was. Hence, the low was labeled as “–”, and the mild and severe were labeled as “+”.

According to immunohistochemical staining for CK19, the condition of DR in marginal areas of nodules was evaluated and subclassified as follows: consecutive = the continuity of DR was intact (positive rate of CK19 > 80%); interrupted = the continuity of DR broke off (positive rate of CK19 between 20% and 80%); absent = the immunohistochemical staining for CK19 was negative or positive sporadically (positive rate of CK19 < 20%). Interruption and absence meant the DR was destroyed, indicating the presence of stromal invasion (Figure 2). Hence, the condition of interruption or absence was labeled as “+”, and consecutiveness as “-”.

The quantities of SAs, medium-power fields (MPFs, 10 × 10) and the intranodule portal area (IPA) were calculated in all sections. The IPA meant a portal area was located within a nodule completely, but not the one that was sandwiched between the lesion and normal liver parenchyma. Eventually, the average densities of SA and IPA were demonstrated in the form of the number of them per 10 MPFs. The number of SAs in one MPF was also evaluated. That meant the distance between two independent SAs was less than a diameter of one MPF (Figure 3). This was another brief method to estimate SA semiquantitatively. The semiquantitative assessments of HSP70, GPC3 and GS were performed as reported previously [6].

### Statistics

SPSS Statistics (Version 22.0, IBM, New York, USA) was used to analyze the data acquired from this study. The data are described as number, percentage, or mean and standard deviation. Continuous variables were analyzed with Student's *t* test or non-parametric test, and they could be converted to categorical variables where appropriate. Categorical variables were compared with the chi-squared test or Fisher's exact test. Normal reference values and receiver operating characteristic (ROC) curve analysis were applied to determine the optimal cut-offs of continuous variables. All *P* values were two tailed and a level of <0.05 was considered to be statistically significant.

## SUPPLEMENTARY MATERIALS TABLES



## Abbreviations

HGDNs: high-grade dysplastic nodules
WD-SHCCs: well-differentiated small hepatocellular carcinomas
LGDNs: low-grade dysplastic nodules
eHCC: early hepatocellular carcinoma
SMCs: stromal morphological changes
NIN: nodule in nodule
HSP70: heat shock protein 70
GPC3: glypican 3
GS: glutamine synthetase
SC: sinusoid capillarization
DR: ductular reaction
SA: solitary artery
CD34: cluster of differentiation 34
CK19: cytokeratin 19
CK7: cytokeratin 7
H&E: Hematoxylin and Eeosin
SHCC-G1: hepatocellular carcinoma grade 1
ROC: receiver operating characteristic
PLT: platelets
PT: prothrombin time
A/G: albumin/globulin
ALT: alanine aminotransferase
AFP: α-fetal protein
CA19-9: carbohydrate antigen19-9
PPV: positive predictive value
NPV: negative predictive value
MPFs: medium-power fields
