# Supplementary material for: The stromal morphological changes for differential diagnosis of uninodular high-grade dysplastic nodule and well-differentiated small hepatocellular carcinoma
Source: Oncotarget. 2017 Sep 1;8(50):87329–39. doi: 10.18632/oncotarget.20607 (PMC5675636; doi:10.18632/oncotarget.20607)
Supplement: Supplementary file 1 [file oncotarget-08-87329-s001.pdf]

The stromal morphological changes for differential diagnosis of uninodular high-grade dysplastic nodule and well-differentiated small hepatocellular carcinoma

SUPPLEMENTARY MATERIALS

Supplementary Table 1: Clinicopathologic data of 31 uninodular HGDNs

No.	Age	Sex	Hepatitis	HBsAg	Size (cm)	AFP (μg/l)	CA19-9 (U/ml)	Cirrhosis	Canceration
1	50	F	HBV	+	2.2	213.4	49.4	Y	N
2	67	F	HBV	+	0.6	3.8	11.5	N	Y
3	39	M	HBV	+	1.5	19.8	61.7	Y	Y
4	56	M	HBV/HCV	+	4.0	17.2	5.0	Y	Y
5	54	M	HBV	+	2.1	56.4	109.8	Y	N
6	50	M	HBV	+	3.0	50.8	43.1	Y	N
7	45	M	HBV	+	2.0	1.5	15.4	N	N
8	59	M	HBV	+	3.8	66.1	109.4	Y	N
9	59	M	HBV	+	2.5	7.3	*	N	N
10	59	M	HBV	+	1.4	5.7	102.1	Y	N
11	56	M	HBV	+	2.1	8.0	1.1	Y	N
12	58	F	HBV	+	2.1	20.2	88.1	Y	Y
13	46	M	HBV	+	1.3	2.3	0.6	Y	N
14	58	M	HBV	+	1.1	144.1	52.1	Y	N
15	58	M	HBV	+	3.7	2.2	0.6	Y	N
16	62	F	HBV	+	4.2	10.9	51.9	N	N
17	57	M	HBV	+	3.0	8.5	75.4	Y	Y
18	65	M	HBV	+	3.5	10.7	72.7	Y	N
19	57	M	HBV	+	5.2	47.9	54.7	Y	N
20	74	M	HCV	–	3.1	2.7	12.2	Y	N
21	71	M	HBV	+	3.3	5.6	46.8	Y	N
22	66	F	HBV	+	1.9	73.9	94.0	Y	Y
23	52	F	HBV	+	2.4	6.0	97.0	Y	Y
24	55	M	HBV	+	4.4	3.1	162.8	Y	Y
25	55	M	HBV	+	2.1	4.8	25.9	Y	Y
26	49	F	HBV	+	1.2	355.9	0.6	Y	Y
27	49	M	HBV	+	2.3	20.2	82.3	N	Y
28	53	F	HBV	+	2.1	3.3	28.1	Y	Y
29	61	F	HBV	+	2.3	43.4	53.3	Y	Y
30	64	M	HBV	+	2.3	6.3	2.2	Y	Y
31	54	M	HBV	+	2.7	2.8	133.4	Y	Y

HGDNs, high-grade dysplastic nodules; F, female; M, male; Y, yes; N, no. Reference range: AFP: 0–20 μg/l; CA19-9, 0–39 U/ml. “*”, Negative but without exact figure.

Supplementary Table 2: Clinicopathologic data of 32 uninodular WD-SHCCs

No.	Age	Sex	Hepatitis	HBsAg	Size (cm)	AFP (µg/l)	CA19-9 (U/ml)	Cirrhosis
1	56	M	HBV	+	2.6	11.2	17.7	N
2	64	F	HBV	+	3	14.7	2.9	N
3	42	M	HBV	+	0.6	7.2	8.5	N
4	51	M	HBV	+	1.4	2.0	23.8	Y
5	69	M	HBV	+	1.5	1.5	43.7	N
6	57	F	HBV	+	3	1.0	1.0	N
7	57	M	HBV	+	2	7.4	5.0	Y
8	43	M	HBV	+	1.3	1.6	3.0	Y
9	68	F	HCV	-	2.4	2.4	1.6	Y
10	66	M	HBV	+	1.1	7.0	24.8	N
11	60	F	HCV	-	3	104.6	212.1	N
12	59	M	HBV	+	2.2	6.4	44.2	Y
13	63	M	HCV	–	3	2.9	10.7	Y
14	58	M	None	–	2.3	2.2	11.2	Y
15	60	M	HBV	+	2.3	5.4	20.4	Y
16	61	F	HBV	+	2.3	10.9	51.5	Y
17	79	M	HBV	-	2.5	2.9	23.1	Y
18	64	F	HBV	+	2.3	8.9	33.3	Y
19	68	M	HBV	+	2.4	11.2	50.5	N
20	46	M	HBV	+	1.7	119.0	0.9	N
21	58	M	None	–	1.9	3.8	15.7	Y
22	49	M	None	–	2	4.4	51.6	N
23	47	M	HBV	+	1.7	3.0	13.6	N
24	50	F	HBV	+	2.7	19.7	13.8	N
25	64	M	HBV	+	2.6	2.0	5.9	Y
26	67	M	HBV	+	2.4	1.9	5.9	Y
27	63	M	HBV	+	2.1	2.9	24.4	N
28	51	M	HBV	+	1.6	2.1	4.2	N
29	46	M	None	–	2	2.6	16.6	N
30	63	M	HBV	+	3	4.1	21.8	Y
31	38	M	HBV	+	3	7.3	0.6	Y
32	58	M	HBV	+	1.3	6.8	30.1	N

WD-SHCCs, well-differentiated small hepatocellular Carcinoma; F, female; M, male; (+), positive; (–), negative; Y, yes; N, no. Reference range: AFP: 0–20 µg/l; CA19-9, 0–39 U/ml.

Supplementary Table 3: The 8 potential combinations of SMCs in HGDNs and WD-SHCCs

Subtype	HGDNs (*n* = 31)	WD-SHCCs (*n* = 32)
SC+/DR+/SV+	0	21 (65.62%)
SC+/DR+/SV–	0	1 (3.13%)
SC+/DR–/SV+	1 (3.23%)	3 (9.38%)
SC+/DR–/SV–	2 (6.45%)	0
SC–/DR+/SV+	2 (6.45%)	5 (15.62%)
SC–/DR+/SV–	4 (12.90%)	1 (3.13%)
SC–/DR–/SV+	2 (6.45%)	1 (3.13%)
SC–/DR–/SV–	20 (64.52%)	0

Abbreviation: SMCs, Stromal morphological changes; WD-SHCCs, well-differentiated small hepatocellular carcinomas; HGDNs, high-grade dysplastic nodules; SC, sinusoid capillarization; DR, ductular reaction; SV, Solitary arteries.

Supplementary Table 4: Diagnostic performance of hematic PLT, AFP and CA19-9 for HGDNs detection

Subgroups	HGDNs (*n* = 31)	WD-SHCCs (*n* = 32)	Sensitivity (%)	Specificity (%)	PPV (%)	NPV (%)	Accuracy (%)
3 indexes							
All 3 positive	8	1	25.81	96.88	88.89	57.41	61.90
At least 2 positive	19	5	61.29	84.38	79.17	69.23	73.02
At least 1 positive	29	16	93.55	50.00	64.44	88.89	71.43
2 indexes							
PLT+/AFP+	9	1	29.03	96.88	90.00	58.49	63.49
AFP+/ CA19-9+	10	1	32.26	96.88	90.91	59.62	65.08
PLT+/CA19-9+	15	5	48.39	84.38	75.00	62.79	66.67
1 index							
PLT+	26	13	83.87	59.38	66.67	79.17	71.43
AFP+	11	2	35.48	93.75	84.63	60.00	65.08
CA19-9+	19	7	61.29	78.13	73.08	67.57	69.84

Abbreviations: WD-SHCCs, well-differentiated small hepatocellular carcinomas; HGDNs, high-grade dysplastic nodules; PPV: positive predictive value; NPV: negative predictive value; PLT, platelets,(+: $< 125 \times 10^9$/L,-: 125–350 $\times 10^9$/L); AFP, alpha fetal protein (+: > 20 μg/l,-: 0–20 μg/l); CA19-9, carbohydrate antigen 19-9 (+: > 39 U/ml,-: 0–39 U/ml).

Supplementary Table 5: Multivariate analysis of SC, DR and SA between HGDNs and WD-SHCCs

Pathological parameters	*P* value	OR
SC	0.013	22.012
DR	0.009	24.991
SV	0.006	22.811

SC, sinusoid capillarization; DR, ductular reaction; SV, solitary vessel; HGDNs, high-grade dysplastic nodules; WD-SHCCs, well-differentiated small hepatocellular carcinomas; OR, Odd ratio.

Supplementary Table 6: The diagnostic performance of imaging for HGDNs and WD-SHCCs

	Typical characteristics of HCC		Total
	Yes	No	
WD-SHCCs	26	3	29
HGDNs	18	7	25
Total	44	10	54

HGDNs, high-grade dysplastic nodules; WD-SHCCs, well-differentiated small hepatocellular carcinomas.
